# Optimization of kefir fermentation with plantain peel addition: effects on composition, microbial viability, and sensory quality

**DOI:** 10.3389/fnut.2025.1740355

**Published:** 2026-02-03

**Authors:** Andrea Pissatto Peres, Cláudia Puerari, Juliana Aparecida Correia Bento, Rafael Alexandre dos Santos Martins, Yasmin Ourives Domingues, Maressa Caldeira Morzelle

**Affiliations:** Department of Food and Nutrition, Federal University of Mato Grosso, Cuiabá, State of Mato Grosso, Brazil

**Keywords:** by-products, Central Composite Rotatable Design, fermented milk, sensory analysis, kefir-based

## Abstract

Kefir is a fermented dairy product that can be prepared through microbial fermentation using kefir grains. These grains consist of a symbiotic community of bacteria and yeasts that influence the chemical composition, texture, and sensory characteristics of fermented milk. The incorporation of fruit by-products during fermentation has been explored as a strategy to enhance the functional quality of kefir-based beverages. Among them, plantain (*Musa paradisiaca*) by-products represent a promising source of bioactive compounds with antioxidant potential and significant amounts of dietary fiber. This study aimed to optimize the fermentation conditions of milk kefir enriched with green plantain peel using response surface methodology and evaluate the microbial viability of the optimized beverage during 21 days of storage. Fermentation parameters were established through preliminary tests, employing UHT milk, sugar (8%), kefir grains, and green plantain peel, fermented for 4 h at 25 °C, using a central composite rotational design (CCRD). The CCRD included two independent variables (X1: green plantain peel 10%−30.0% and X_2_: Kefir grains, 5%−20%). The optimized formulation, containing 20% green plantain peel and 10% kefir grains, showed increased protein content and reduced carbohydrate levels compared to the control beverage. Although higher inoculum levels did not significantly enhance bioactive compound content, this was likely due to microbial utilization of these metabolites. Lactic acid bacteria (LAB) counts increased over storage, reaching ~104 CFU ml^−1^ after 21 days, demonstrating the stability of the core kefir microbiota. Sensory evaluation indicated an overall acceptability index of 81.29%. In conclusion, the enrichment of milk kefir with green plantain peel resulted in a nutritionally improved and sensorially accepted beverage, characterized by higher protein density and lower carbohydrate content. These findings highlight the potential of plantain peel as a functional ingredient for the development of enhanced fermented dairy products.

## 1 Introduction

Milk kefir is a fermented dairy beverage produced through the activity of a complex symbiotic community of microorganisms present in kefir grains, predominantly including lactic acid bacteria (LAB), acetic acid bacteria (AAB), and yeasts. This microbial community interacts synergistically, which is fundamental in imparting kefir with its distinct sensory profile and valuable functional properties. Structurally, Kefir grains are mainly composed of proteins, lipids, and the soluble exopolysaccharide kefiran, which plays a structural role in maintaining the grain's integrity and contributes to the texture and viscosity of the final product (1).

Milk, as the substrate for kefir fermentation, is a nutritionally dense food, rich in high-quality protein, calcium, essential vitamins (notably B2, B12, A, D), and important minerals such as phosphorus, potassium, magnesium, and zinc, in addition to containing saturated and unsaturated fats and lactoferrin, immunoglobulins, and enzymes (2, 3).

In the global functional food market, products containing probiotics, such as kefir, account for ~60% (4). Probiotic beverages like kefir serve as efficient delivery systems for beneficial microorganisms, mainly from the *Lactobacillaceae* and *Bifidobacteriaceae* families, which are capable of competing with enteric pathogens, including *Escherichia coli* and *Clostridium perfringens*. When these formulations are combined with prebiotic substrates, they can exhibit synergistic (symbiotic) effects that enhance microbial viability and optimize physiological outcomes, such as immune modulation, antimicrobial activity, sleep quality, and gut microbiota balance. Thus, the integration of prebiotic-rich ingredients into fermented beverages constitutes an effective strategy to amplify their functional and health-promoting potentials (5, 6).

Among the potential prebiotic sources, plantain (*Musa paradisiaca*) stands out for its nutritional composition and availability. This fruit, widely cultivated and consumed in tropical regions, is an integral part of local diets and food cultures, particularly in Latin America and Africa (7). The peel of the plantain which accounts for about 40% of the fruit's weight, and is frequently discarded as waste. However, this by-product contains high levels of dietary fiber, minerals, phenolic compounds, and other phytochemicals with antioxidant and antimicrobial properties (8–10). These bioactive compounds confer the peel a promising potential on the peel as a functional ingredient for enriching food products, especially fermented dairy matrices.

Recent studies have shown that fruit peels and other plant-based by-products can promote the growth and stability of lactic acid bacteria, helping to maintain viable counts above 7 log CFU g^−1^ or ml^−1^ during storage. This effect is attributed to the presence of phenolic compounds and fibers that can act as prebiotic substrates, stimulating the viability and functionality of microorganisms (11–15). The co-fermentation of milk with fruits or by-products, namely peels and seeds rich in polyphenols, vitamins, minerals, and other dietary fiber, has been reported as a way to enhance the nutritional profile and biological activity of fermented foods (16). Furthermore, the valorization of these agro-industrial residues aligns with sustainable food production models and the principles of the circular economy, directly contributing to the United Nations Sustainable Development Goals (SDGs), by reducing food waste, improving resource efficiency, and promoting environmental preservation (17–19).

In this context, the incorporation of green plantain peel into milk kefir emerges as an innovative approach for developing a functional fermented beverage that not only improves the nutritional and functional quality of kefir but also supports waste reduction and sustainability. It is noteworthy that, to date, there are no published scientific reports detailing the optimization of a fermented beverage incorporating plantain peel. Therefore, the main objective of the present study was to evaluate, through the optimization and empirical modeling of fermentation conditions using response surface methodology (CCRD), the feasibility in incorporating green plantain peel into fermented milk with kefir. Additionally, the study sought to characterize the physicochemical properties, microbiological viability during 21 days of storage, and sensory acceptance of the optimized formulation.

## 2 Material and methods

### 2.1 Raw materials collection

The UHT milk was purchased from a local store and the plantain peel was donated by a producer of artisanal fried plantains (T.A Alimentos). Green plantain peel (GPP) classified using the Von Loesecke escale—value 2 (20), was selected, washed in clean water and then sanitized with sodium hypochlorite (NaClO 1%; p/v) for 15 min (21). Then the peel was cut and cooked in water (60% v/v) and maintained at 80 °C for 10 min and frozen at −18 °C until use. Its proximal composition (%) was moisture (85.0 ± 0.15), ash (1.48 ± 0.17), protein (6.22 ± 0.12), lipids (0.34 ± 0.05), carbohydrate (6.96), insoluble fiber (2.7), and soluble fiber (0.55).

### 2.2 Kefir

Milk kefir grains, from the culture bank of the Bioprocess and Fermentative Process Laboratory, Department of Food and Nutrition, Federal University of Mato Grosso, were activated for 30 days before fermentation using milk changed daily. Preliminary tests established the 4 h at 25 °C condition to achieve the targeted optimum fermentation parameters of pH < 4.5 and acidity between 0.6 and 2.0 g of lactic acid 100 g^−1^. The grains were kept at 8 °C until the next fermentation.

### 2.3 Fermentation conditions–Central Composite Rotational Design (CCRD)

The experiment consisted of two stages: (1) optimization of the beverage production process using a Central Composite Rotatable Design (CCRD), (2) Physical and chemical characterization, probiotic viability, and sensory quality of the optimized kefir-based beverage (KPP).

The CCRD consisted of two variables (X1: green plantain peel, 10.0 a 30.0% and X_2_: Kefir grains, 5.0 a 20.0%; Table 1). For fermentation processes, UHT whole milk, sugar (8%), kefir grains, and green plantain peel, were used. The fermentation time was 4 h at 25 °C (established by preliminary tests) to achieve the optimum fermentation conditions to reach pH < 4.5 and acidity between 0.6 and 2.0 g of lactic acid 100 g^−1^ (22). The samples were cooled to 8 °C and set aside for analysis in triplicate.

**Table 1:** Experimental matrix of the central composite rotatable design (CCRD) for milk kefir formulation, detailing encoded and uncoded real values.

**Run**	**Encoded variables**		**Uncoded variables**	
	**x1**	**x2**	**Green plantain peel (%)**	**Kefir grains (%)**
1	1	1	27.0	18.0
2	1	−1	27.0	7.0
3	−1	1	13.0	18.0
4	−1	−1	13.0	7.0
5	1.41	0	30.0	13.0
6	−1.41	0	10.0	13.0
7	0	1.41	20.0	20.0
8	0	−1.41	20.0	5.0
9	0	0	20.0	13.0
10	0	0	20.0	12.5
11	0	0	20.0	12.5
12	0	0	20.0	12.5
13	0	0	20.0	12.5

x1, green plantain peel (GPP, % w/w); x2, kefir grains (% w/w).

The optimized kefir-based beverage was submitted to syneresis, total soluble solids, and water retention capacity (WRC), color, instrumental texture profile analysis, proximal composition, microbiological analysis, microbial viability, and sensory evaluation.

### 2.4 Physicochemical evaluation

The pH, total titratable acidity (TTA), and soluble solids (°Brix) of the optimized kefir-based beverage were determined following standardized analytical protocols. The pH was measured using a digital potentiometer (Ms Tecnopon Instrumentação, Mpa-210, Brazil) previously calibrated with pH 4.0 and 7.0 buffer solutions (23). TTA was determined by titration with 0.1 M sodium hydroxide (NaOH) until pH 8.2–8.4, as described ISO (23). Results were expressed as a percentage of lactic acid (% w/w). Soluble solids were quantified using an Abbé refractometer. Analyses were conducted on optimized kefir-based beverage (KPP) after 4 h fermentation.

### 2.5 Total phenolic compounds (TPC) and antioxidant activity (FRAP, DPPH and ABTS)

The total phenolic compounds were quantified in triplicate using the Folin-Ciocalteu reagent method, according to the protocol described by Woisky and Salatino (24). The absorbance was measured using a spectrophotometer (Biochrom, model Libra S32, Cambridge, England) at 740 nm. TPC were quantified by constructing a standard curve using gallic acid with concentrations ranging from 0 to 100 μM. The results were expressed in μg of gallic acid equivalents (GAE) per gram of sample.

The evaluation of antioxidant activity using the DPPH method followed the procedures described by Brand-Williams et al. (25). The absorbances were measured at 517 nm using a spectrophotometer (Biochrom, model Libra S32, Cambridge, England). A standard curve was constructed (200, 400, 600, 800, 1,000 μM) using a reference antioxidant (Trolox) to quantify antioxidant activity. The results were expressed in Trolox equivalents (μM Trolox) per gram of sample. The antioxidant capacity by elimination of the ABTS radical was estimated in triplicate according to the method proposed by Nenadis et al. (26) with modifications. An aliquot of 30 μl of the extract was mixed and homogenized with 3 ml of the ABTS radical solution in test tubes protected from light. After 6 min, the absorbance was measured at 734 nm. The results were expressed as Trolox equivalents (μM Trolox) per gram of sample. The antioxidant potential of FRAP was determined according to the methodology proposed by Pulido et al. (27). The absorbance was measured in a spectrophotometer at 595 nm. The results were calculated based on a standard curve of ferrous sulfate (0–2,000 μM) and expressed in μM of ferrous sulfate per gram of sample. All samples were analyzed in triplicate, and data were presented as mean ± standard deviation.

### 2.6 Syneresis and water retention capacity (WRC)

The syneresis and water retention capacity (WRC) were evaluated according to Mohamed-Ahmed et al. (28) by centrifuging 10 g samples at 5,000 rpm for 10 min at 4 °C. The syneresis and WRC percentage were determined according to Equations 1 and 2.


$$\begin{array}{c} Syneresis\;\left(\%\right)=\frac{whey\;separeted\;\left(g\right)}{sample\;\left(g\right)}*100 \end{array}$$ (1)

$$\begin{array}{c} WRC\;\left(\%\right)\;=\frac{\;precipitated\;gel\;\left(g\right)}{sample\;\left(g\right)}*100 \end{array}$$ (2)

### 2.7 Color analysis

The instrumental color parameters were directly read in a Minolta CR-300 colorimeter (Minolta Camera Co., Osaka, Japan). The color elements (L^*^, a^*^, and b^*^): luminosity (L^*^), and chromaticity coordinates a^*^ and b^*^. The values of L^*^, a^*^, and b^*^ were used to calculate total color difference (ΔE^*^), chroma (C^*^), and Hue angle (H°), using Equations 3–5, respectively (29).


$$\begin{array}{c} \text{Δ}{\text{E}}^{*}=\sqrt{{\left(\text{Δ}{\text{L}}^{*}\right)}^{2}+{\left(\text{Δ}{\text{a}}^{*}\right)}^{2}+{\left(\text{Δ}{\text{b}}^{*}\right)}^{2}} \end{array}$$ (3)

$$\begin{array}{c} {\text{C}}^{*}=\sqrt{({\text{a}}^{*2}+{\text{b}}^{*2})} \end{array}$$ (4)

$$\begin{array}{c} {\text{H}}^{⚬}=\text{arc tg}\left(\frac{{\text{b}}^{\text{u}}}{{\text{a}}^{\text{u}}}\right) \end{array}$$ (5)

### 2.8 Instrumental texture

The P texture of de optimized beverage (KPP) was determined in triplicate after 24 h of refrigerated storage (4 ± 1 °C) in terms of the following parameters: firmness, consistency, cohesiveness, and viscosity index with a TA-XTplus Stable Micro System (Stable Micro Systems, Haslemere, UK). A single penetration test was employed on 60 ml samples contained in individual plastic pots, at 9 ± 2 °C, by a 20 mm diameter acrylic cylinder probe (P/20P). Trigger force: 10 g. Pre-test speed was fixed at 1 mm s^−1^, and post-test speed at 5 mm s^−1^, and the penetration depth was 5 mm (30).

### 2.9 Proximal composition

Moisture content (AOAC 925.45b), fat (AOAC 920.39), protein (AOAC 960.52), and ash (AOAC 923.03) were determined in KPP according to the methodology proposed by AOAC (31). All analyses were performed in triplicate (32), and results were expressed as g.100 g^−1^ on a wet basis. The concentration of carbohydrates was determined by the difference: 100–(% moisture + % protein + % fat + % ash content), and the energy value was calculated using the Atwater method (32).

### 2.10 Microbial viability during storage

Microbial viability was assessed throughout the storage period of the optimized kefir-based beverage. Acetic acid bacteria (AAB) were enumerated by spread-plating on acetic acid medium (AAM) agar supplemented with nystatin (4,000 UI ml^−1^), and incubated aerobically at 28 °C for 7 days (33). Lactic acid bacteria (LAB) counts were determined on Man, Rogosa, and Sharpe (MRS) agar containing nystatin (4,000 UI ml^−1^), incubated under anaerobic conditions at 30 °C for 72 h (34). Yeasts were enumerated on Sabouraud agar supplemented with chloramphenicol (500 mg L^−1^), under aerobic conditions at 30 °C for 120 h (34). The results were obtained as logarithms of the number of colony-forming units ml^−1^ (log CFU ml^−1^). Analyses were performed in triplicate at four storage time points: day 1, day 7, day 14, and day 21 of refrigerated storage.

### 2.11 Microbiological analysis

Before sensory evaluation, microbiological safety analyses were conducted to verify compliance with Brazilian sanitary standards. Samples were serially diluted (1:10) in 0.1% saline peptone water (SPW), and aliquots (1 ml) were spread-plated for enumeration of *Escherichia coli* according to the procedures described by the Brazilian legislation (35). The detection of *Salmonella spp*. was performed following the described by International Organization for Standardization protocol (36). Results were expressed as logarithms of the number of colony-forming units per ml of fermented milk (log CFU ml^−1^) (35).

### 2.12 Sensory evaluation

The sensory evaluation of the optimized kefir-based beverage was conducted with a panel of 114 untrained tasters, including males and females, aged between 18 and 60 years old. The tests took place in the Sensory Analysis Laboratory at FANUT-UFMT, in isolated booths, with ideal lighting and no interferences such as odors or noise. Tasters received randomized, refrigerated (10 °C) 15 ml samples served in plastic cups coded with three random digits and presented monadically, along with a glass of water for palate cleansing. Participants evaluated the samples using a nine-point hedonic scale (9 = like extremely; 1 = dislike extremely) based on the attributes of taste, odor, and appearance. In addition, a purchase intention test was carried out using a structured five-point scale, ranging from “certainly would buy it” to “certainly would not buy it” (37). The acceptability index (AI %) was calculated using the following formula:


$$\begin{array}{c} Acceptabiliy\;index\;\left(\%\right)=\frac{A\;\left(mean\;score\right)}{B\;\left(maximum\;score\right)}*100 \end{array}$$

where *A* represents the mean score obtained for the product and *B* the maximum possible score on the hedonic scale. Samples with an AI ≥ 70% were considered to have good consumer acceptance (37). The sensory evaluation procedures followed ethical standards and were approved by E Ethics Research Committee of the Federal University of Mato Grosso (CAAE: 77988123.3.0000.8124).

### 2.13 Statistical evaluation

The response surface graphs and functions for pH, acidity, total phenolic compounds, and antioxidant activity were generated to define the optimal parameters for fermentation using software STATISTICA^®^ 10.0. Models were validated in three-replicate assays using the optimized formulation. For the quantitative results obtained in the characterization analyses of the optimized beverage, analysis of variance (ANOVA) was performed, followed by Tukey's mean comparison test (*p* < 0.05).

## 3 Results and discussion

### 3.1 Optimization of kefir-based beverage–central composite rotatable design (CCRD)

**Figure 1:**
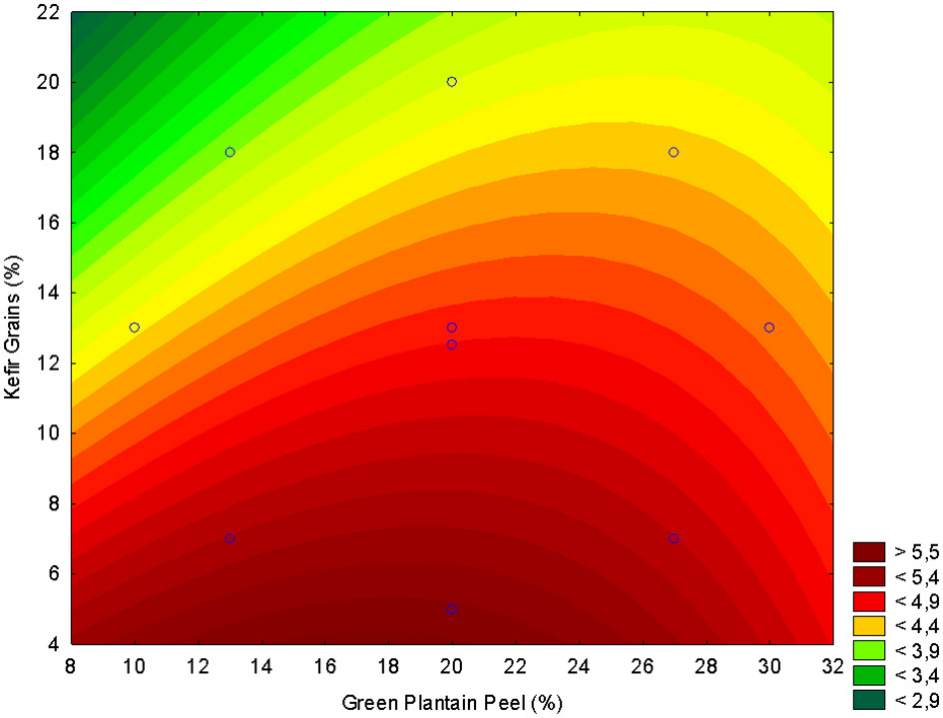
Response surface plot illustrating the effect of green plantain peel concentration (GPP, X_1_) and kefir grain concentration (X_2_) on the final pH of the fermented beverage.

**Figure 2:**
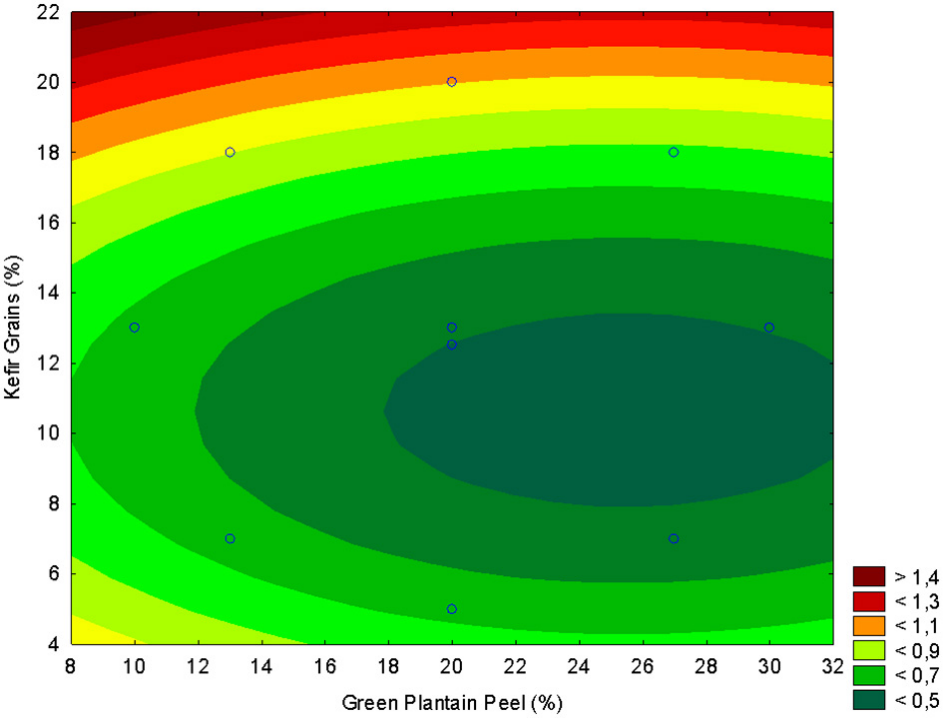
Response surface plot illustrating the effect of green plantain peel concentration (GPP, X_1_) and kefir grain concentration (X_2_) on the Total Titratable Acidity (TTA, %) of the fermented beverage.

**Figure 3:**
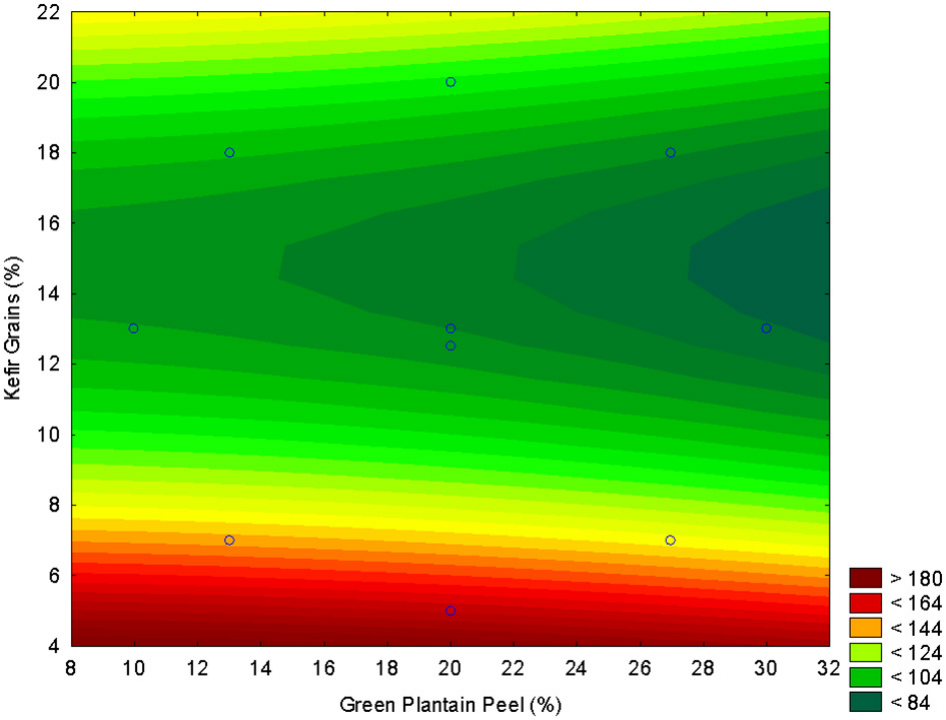
Response surface plot illustrating the effect of green plantain peel concentration (GPP, X_1_) and kefir grain concentration (X_2_) on the Total Phenolic Compounds (TPC) content (μg GAE g^−1^) of the fermented beverage.

**Figure 4:**
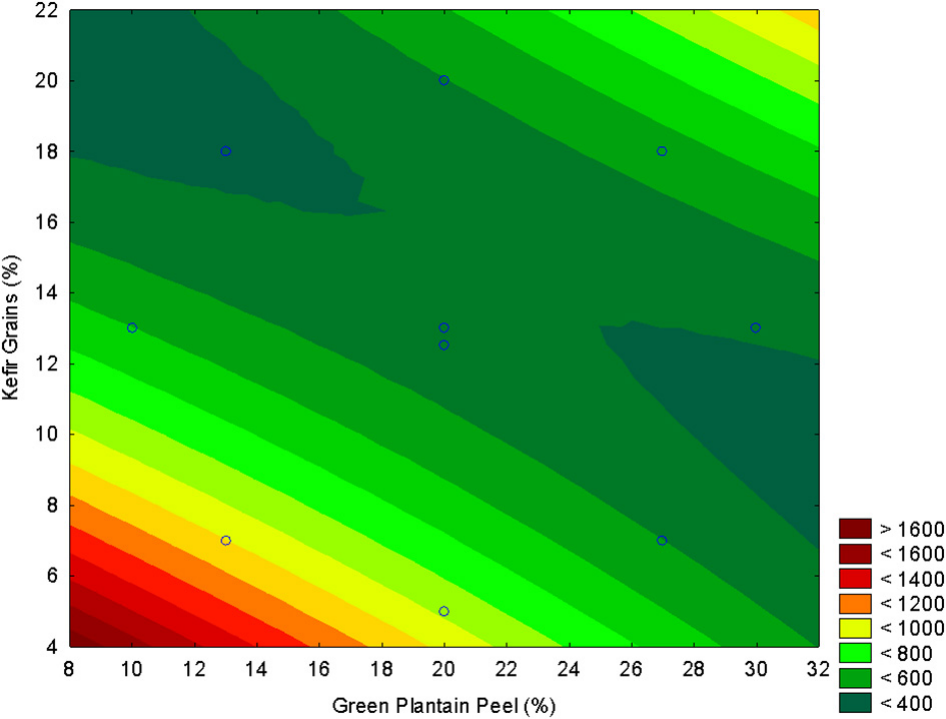
Response surface plots illustrating the effect of green plantain peel concentration (GPP, *X*_1_) and kefir grain concentration (*X*_2_) on the Antioxidant Activity measured by FRAP (μM ferrous sulfate g^−1^).

**Figure 5:**
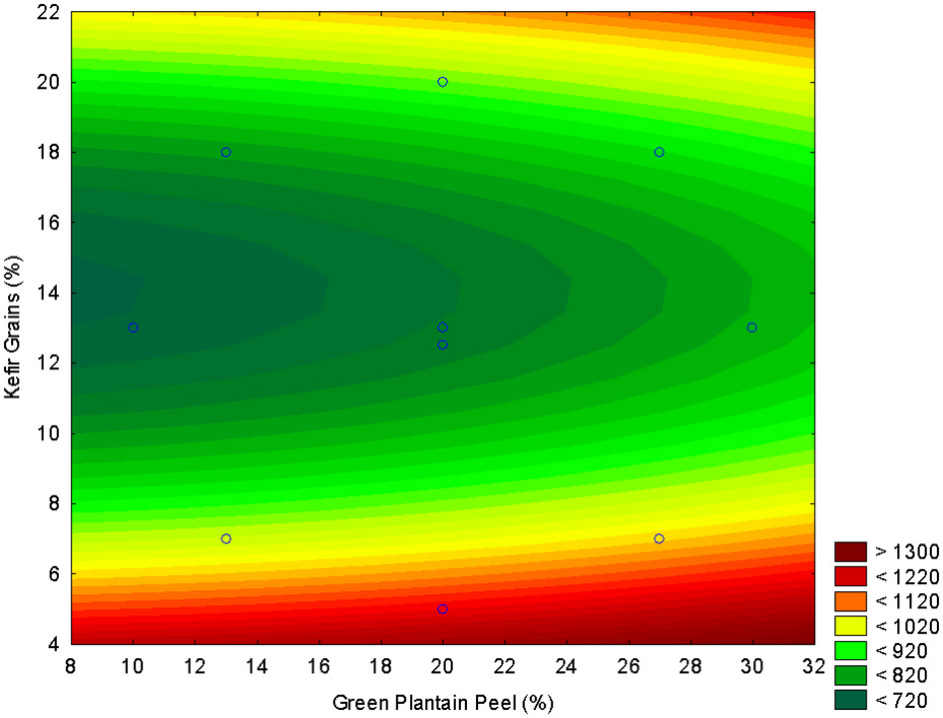
Response surface plots illustrating the effect of green plantain peel concentration (GPP, *X*_1_) and kefir grain concentration (*X*_2_) on the antioxidant activity measured by ABTS (μM Trolox g^−1^).

The experimental responses for pH, TTA, TPC, and antioxidant activity (FRAP, and ABTS) obtained from the CCRD are presented in Figures 1–5. The corresponding second-order regression models are summarized in Table 2. For pH, the fitted model indicated a positive linear and negative quadratic effect for the green plantain peel and a negative linear and positive quadratic effect of kefir grains (Table 2), indicating that increasing the concentration of kefir grains reduces the pH of the beverage (Figure 1). Similar findings are reported with UHT milk, where higher kefir grain concentrations led to a greater reduction in pH and changes in other fermentation by-products. Adding more kefir grains introduces a higher concentration of lactic acid bacteria and yeasts. These microorganisms rapidly ferment sugars, converting them into lactic acid and, to a lesser extent, ethanol and other acids. The accumulation of lactic acid is the primary reason for the drop in pH, and is responsible for making milk acidic (38).

**Table 2:** Second-order regression models (quadratic equations) adjusted for physicochemical and functional responses using the central composite rotatable design (CCRD).

**Experimental data**	**Model**	***R* ^2^**	***R* ajust**	***p*-Value**	**Lack of fit**
pH	*y* = 5.25 + 0.11 x_1_−0.003x_1_^2^−0.168 x_2_ + 0.004x_2_^2^	0.86	0.85	< 0.008	0.00
Total titratable acidity (TTA; %)	*y* = 1.65–0.04 x_1_ + 0.001x_1_^2^−0.128 x_2_ + 0.006 x_2_^2^	0.75	0.73	< 0.001	0.00
TPC (μg GAE por g^−1^)	*y* = 272.6–0.014 x_1_^2^−23.9 x_2_ + 0.81 x_2_^2^	0.57	0.54	< 0.02	0.00
FRAP (μM ferrous sulfate g^−1^)	*y* = 1.722.6 + 0.126 x_1_^2^−144.6 x_2_ + 5.18 x_2_^2^	0.84	0.83	< 0.001	0.92
ABTS (μM trolox g^−1^)	*y* = 3.083.3–97 x_1_ + 0.76 x_1_^2^−223 x_2_ + 4.39 x_2_^2^ + 4.38 x_1_x_2_	0.85	0.83	< 0.001	0.00

x_1_, green plantain peel (g 100 g^−1^); x_2_, kefir grains (ml 100 g^−1^).

Conversely, GPP exhibited a higher intrinsic pH (less acidic), which decreases as the fruit ripens. The pH of the green plantain peel in the study was 5.86 ± 0.15, an approximate value found by Adi et al. (39) of 6.18. As a result, the addition of green plantain peel was made, and kept at 8 °C.

The GPP did not favor the reduction of pH. This is because the compounds in the plantain peel, such as complex carbohydrates, minerals, and alkaline substances, can buffer or neutralize the acids produced during fermentation, causing difficulty in maintaining the pH close to 4.5. Although LAB normally reduces the pH of milk during fermentation by producing lactic acid, the addition of plantain co-product can neutralize this acidification to a certain extent, depending on the amount used (9).

The best concentration was 20% of green plantain peel and 10% kefir grains (Figure 1), giving the best pH result for the fermented beverage around 4.5.

In the titratable acidity analysis, the adjusted model showed a negative linear and positive quadratic effect for green plantain peel (X1) and a negative linear and positive quadratic effect for kefir grains X_2_ (Table 2). Therefore, the addition of plantain peel did not significantly influence the acidity of the fermented beverage, and it was inversely proportional to the results observed in the pH assessment.

The best concentration of green plantain peel was 20%−26% and 5%−10% for kefir grains (Figure 2).

The regression model adjusted for Total Phenolic Compounds (TPC) showed a negative quadratic effect for green plantain peel, and a negative linear and positive quadratic effect for the kefir grains (Table 2). Even with the increase of kefir grains in the fermented beverage, there was no increase in bioactive compounds with the addition of plantain peel, and suggesting consumption of bioactive compounds by microorganisms. Therefore, the lower the addition of kefir grains, better the correlation with bioactive compounds (Figure 3). This supports that, while fermentation and extraction methods can enhance the phenolic content of fruit peels, the resulting phenolic profile and bioaccessibility are contingent upon the specific peel type, fermentation parameters, and the extraction technique employed. This variability may account for the observation that the addition of green plantain peel does not consistently lead to a significant alteration in the phenolic levels of a fermented beverage (40–42).

In agreement with TPC, the same behavior was observed for FRAP (Figure 4). The regression model adjusted for FRAP showed a positive quadratic effect for green plantain peel, a negative linear effect, and a positive quadratic effect for kefir grains (Table 2).

The increase in the concentration of kefir grains in the production of the beverage reduced its oxidizing potential. The capacity of plantain peel to oxidize metal ions, such as Fe, since antioxidant activity was not detected in the FRAP method. The observed result can be attributed to the high mineral content of plantain peel, which is rich in trace elements such as zinc (Zn) and copper (Cu). These elements are inversely correlated with the results of the Ferric Reducing Antioxidant Power (FRAP). Furthermore, studies show that increasing the concentration of kefir grains during fermentation leads to higher antioxidant activity and a greater reduction in oxidizing potential in various plant-based and dairy beverages. This is typically measured by increased radical scavenging activity (DPPH, ABTS), reducing power, and total phenolic content (43–45).

As demonstrated in Figure 5, the addition of plantain peel in quantities exceeding 20% results in a decrease in the antioxidant activity, as measured by ABTS, of the kefir-based beverage. Consequently, the incorporation of more than 20% green plantain peel into milk kefir is not recommended if the objective is to achieve a beverage with enhanced antioxidant activity. Fermentation often leads to a decrease in total phenolic content (TPC) and total flavonoid content (TFC), which are key contributors to antioxidant activity. Microbial enzymes can break down these compounds, resulting in lower antioxidant potential in the final product. And then, fungi and bacteria used in fermentation can consume phenolic compounds as substrates, further reducing their concentration and thus the antioxidant activity (15, 46).

According to the results presented, and to obtain milk kefir with a greater supply of phenolic compounds and/or antioxidant activity, and with a pH lower than 4.5, the formulation of 20% green plantain peel and 10% kefir grains was chosen to optimize multiple responses, including TPC, antioxidant activity, and pH < 4.5. For instance, reducing the grain concentration (from 18% to 10%) yielded better relationships with bioactive compounds.

#### 3.1.1 Experimental validation

After determining the best treatment, a new experiment was conducted. The optimized kefir-based beverage was reevaluated for pH, acidity, total phenolic compounds, and antioxidant activity using FRAP and ABTS to validate the mathematical models obtained using the response surface. For all variables, the value estimated by the model was calculated. The model error was obtained based on the value obtained in the reanalysis of the optimized beverage (Table 3).

**Table 3:** Experimental validation of the regression models for physicochemical and antioxidant responses in the optimized kefir-based beverage (KPP).

**Variables**	**Optimized values**	**Estimated value**	**Real value**	**% error**
pH	X_1_ = 20; X_2_ = 10	4.61	4.56 ± 0.09	1.08
TTA (%)	X_1_ = 20; X_2_ = 10	0.57	0.59 ± 0.05	12.28
TPC (μg GAE por g^−1^)	X_1_ = 20; X_2_ = 10	109.00	61.12 ± 3.63	43.92
FRAP (μM ferrous sulfate g^−1^)	X_1_ = 20; X_2_ = 10	845.00	992.00 ± 15.15	17.39
ABTS (μM trolox g^−1^)	X_1_ = 20; X_2_ = 10	532.30	624.17 ± 48.57	17.26

Optimized conditions tested: X_1_ = 20% GPP; X_2_ = 10% kefir grains.

The pH value showed the lowest error among all variables evaluated, demonstrating high accuracy of the mathematical model for this parameter. The variation observed remained within the standard deviation, indicating good predictive capacity and stability of the product in relation to pH. For acidity, the error was higher than that of pH, although it remains within an acceptable limit, considering the variability inherent in fermentation processes. The model predicted slightly higher acidity levels than those observed experimentally, possibly due to microbiological changes or variation in the availability of sugars and acidic compounds present in the plantain peel.

The observed difference between the predicted optimal TTA and the validation result is a classic and expected outcome when applying statistical modeling to inherently variable biological fermentation processes. Considering that Kefir is mediated by a complex, symbiotic microbial community, and the metabolic activity of the specific grain batch used for the validation run can slightly differ from the average activity modeled in the CCDR, impacting the overall rate of lactic acid production (47). Furthermore, the nutritional composition of the milk substrate, particularly the lactose and available growth factors, is subject to natural seasonal or batch variations, which directly influence the growth and acidification kinetics of the lactic acid bacteria (48). Therefore, the 0.59 g 100 g^−1^ TTA represents the real-world fermentation outcome, which falls within an acceptable biological error margin for this complex probiotic product, compared to the statistical prediction.

For total phenolic compound (TPC) content, the largest error was found among the variables, with a tendency for overestimation by the model. This result suggests that the polynomial model derived from the response surface is a mathematical simplification that was inadequate to accurately represent the complex, non-linear kinetics of the microbial fermentation process.

This poor model fit may be associated with factors such as fermentation time or, as previously discussed, the hypothesis that kefir grains consumed part of these compounds, reducing their concentration. For FRAP antioxidant potential, the model underestimated the actual values, suggesting that the synergy between the bioactive compounds from kefir and the phenolics from plantain was more significant than predicted. Similarly, the experimentally observed ABTS values were higher than predicted, suggesting that the fermented system may have produced additional antioxidant compounds, such as bioactive peptides or organic acids, that were not fully considered in the predictions. The addition of green plantain peel (GPP) to kefir-based beverages can alter antioxidant assay results in complex ways. Understanding why FRAP increases while DPPH decreases requires examining the chemical nature of GPP-derived compounds and the principles of each assay.

The FRAP assay measures the ability of antioxidants to reduce Fe3+ to Fe2+. GPP is rich in phenolic compounds and other reducing agents, which can be released or transformed during fermentation. These compounds, especially after microbial biotransformation, may have strong electron-donating capacity, directly increasing FRAP values by efficiently reducing ferric ions (49). Similar effects have been observed with other fruit peels and plant extracts added to kefir, where fermentation enhances the release and activity of phenolics, boosting FRAP (50).

The DPPH assay measures the ability to scavenge a stable free radical via hydrogen atom or electron donation. Not all phenolic compounds or fermentation products are equally effective in this assay. Some antioxidants generated or released from GPP during fermentation may have limited hydrogen-donating ability or may react more slowly with DPPH, leading to a decrease in measured DPPH activity despite an overall increase in reducing power. Additionally, fermentation can degrade or transform certain antioxidants that are highly active in the DPPH assay, further reducing DPPH values (51).

Furthermore, the increase in FRAP and decrease in DPPH after GPP addition to kefir likely result from the selective release and transformation of antioxidant compounds during fermentation, favoring those with strong reducing power over those with DPPH radical scavenging activity. This underscores the importance of using multiple assays to fully characterize antioxidant changes in functional beverages (14).

### 3.2 Technological characterization of the optimized kefir-based beverage (KPP)

Table 4 shows the syneresis and the WRC of the control and the KPP. The optimized kefir-based beverage exhibited a syneresis value of 60.7% ± 0.38, which was slightly higher than that reported by (15) for yogurt formulated with 20% plantain peel (57%). This increase can be explained by the fundamental differences in their microbial composition and the resulting structure of the milk protein gel, since the presence of yeasts and acetic acid bacteria contributes to a weaker, less compact protein network (physical disruption of the gel) and a higher degree of proteolysis which lead to a weaker and less stable protein network unable to efficiently hold the whey (52, 53). Despite this, the observed value still indicates an acceptable stability for a kefir beverage, as moderate syneresis is common in fermented systems containing plant components. Optimizing formulations aims to minimize syneresis and improve texture stability, contributing to consumer acceptance. Moreover, the water retention capacity of 39.3 ± 0.38% suggests a relatively cohesive gel structure with reduced whey separation, which is desirable for maintaining the physical stability and sensory quality of the product, maintained a favorable mouthfeel, thus contributing to the high overall acceptability index of 81.29% recorded in the sensory analysis (54, 55).

**Table 4:** Comparison of technological properties (syneresis, water retention capacity, color, and instrumental texture) between the control kefir (KC) and the optimized kefir-based beverage (KPP).

**Variable**	**Kefir-based beverage–control (KC)**	**Optimized kefir-based beverage (KPP)**
Syneresis (%)	56.90 ± 1.85^b^	60.70 ± 0.38^a^
Water retention capacity (WRC)	43.10 ± 1.85^a^	39.30 ± 0.38^a^
L	78.99 ± 2.22^a^	79.25 ± 1.22^a^
a^*^	−6.79 ± 1.12^a^	−5.78 ± 0.76^a^
b^*^	9.80 ± 0.52^b^	11.48 ± 0.86^a^
Firmness (g)	9.79 ± 0.39^b^	12.52 ± 0.64^a^
Cohesiveness (g)	2.41 ± 0.12^a^	3.05 ± 0.91^a^
Consistency (g.s)	77.51 ± 1.84^a^	72.70 ± 3.38^a^
Viscosity index (g.s)	61.72 ± 2.41^a^	50.00 ± 2.30^b^

Different letters (a, b) in the line indicate a statistical difference between the means using the Tukey test (*p* < 0.05).

Dietary fibers from fruit peels, including plantain and banana, typically bind water and reinforce the gel matrix, thereby reducing syneresis in fermented dairy products such as yogurt and kefir. This phenomenon can be attributed to the capacity of fibers to entrap water within the protein network, thereby stabilizing the gel and preventing whey separation (56).

However, it has been demonstrated that excessive fiber intake can disrupt the protein network. In the event of elevated levels of fiber, the gel structure may be compromised, resulting in diminished water-holding capacity and heightened syneresis. This strange effect occurs because the fiber interferes with protein–protein interactions, resulting in a less cohesive gel. Plantain peel has been found to be rich in insoluble fiber, which is composed of cellulose, hemicellulose and lignin. This fiber has the capacity to physically disrupt the casein network if it is not incorporated in an optimal manner. This disruption has the potential to increase syneresis in certain formulations (57).

In terms of color, the only component that showed a statistical difference was component b^*^, with a higher value in the KPP, indicating a more yellowish color (Table 4). Regarding color parameters, enriched with plantain peel resulted in lower luminosity (L^*^), which can be explained by the high content of dark pigments in the peel. Chroma (C^*^) represents the intensity or saturation of color, while the hue angle (h°) defines the perceived tone (58). The optimized kefir-based beverage showed a very pale greenish-yellow hue (h° 160–170) with low chroma values (C^*^ < 10), indicating subtle coloration.

In the instrumental texture parameters, it was found that the optimized beverage presented the highest value for firmness and the lowest viscosity index. The cohesion and consistency values were statistically equal (Table 4). When plantain peel is added in significant amounts, it may disrupt the continuous protein network formed during milk fermentation, leading to a less cohesive gel and thus lower viscosity to physical interference and dilution effects. During milk fermentation, proteins (mainly casein) form a continuous gel network that gives kefir its viscosity and texture. When insoluble dietary fibers like plantain peel are added, they can physically interfere with the formation of this network, acting as inert fillers that prevent proteins from cross-linking efficiently. This results in a weaker, less cohesive gel and reduced viscosity (59). Excess fiber can also act as a filler, diluting the protein matrix and reducing the beverage's viscosity (60).

### 3.3 Proximal composition, total phenolic compounds (TPC), and antioxidant activity (FRAP, DPPH, ABTS) of the optimized kefir-based beverage

Table 5 presents the proximal composition, total phenolic compounds (TPC), and antioxidant activity (FRAP, DPPH, ABTS) of the optimized kefir-based beverage (KPP) and control (KC).

**Table 5:** Proximal composition, total phenolic compounds (TPC), and antioxidant activity (FRAP, DPPH, ABTS) of the kefir-based control (KC) and the optimized kefir-based beverage (KPP).

**Components**	**Kefir-based control (KC)**	**Optimized kefir-based beverage (KPP)**
Total soluble solids (°Brix)	15.00 ± 0.50^a^	10.5 ± 0.86^b^
Moisture (g.100 g^−1^)	82.27 ± 0.14^b^	84.58 ± 0.02^a^
Ash (g.100 g^−1^)	0.59 ± 0.08^a^	0.55 ± 0.11^a^
Lipids (g.100 g^−1^)	2.50 ± 0.25^a^	2.33 ± 0.25^a^
Protein (g.100 g^−1^)	2.35 ± 0.01^b^	3.07 ± 0.18^a^
Total carbohydrate ^1^ (g.100 g^−1^)	12.29	9.47
Energetic value (Kcal.100 g^−1^)	81.06	71.13
TPC (μg GAE por g^−1^)	109.38 ± 2.70^a^	61.12 ± 3,63^b^
FRAP (μM ferrous sulfate g^−1^)	870.33 ± 18.39^b^	992.00 ± 15.15^a^
DPPH (μM trolox g^−1^)	2,485.83 ± 14.30^a^	1,018.2 ± 18.29^b^
ABTS (μM trolox g^−1^)	397.69 ± 6.38^b^	624.17 ± 48.57^a^

Different letters (a, b) in the column indicate a statistical difference between the means using the Tukey test (*p* < 0.05).

Total Carbohydrate determined by difference [100–(Moisture + Protein + Fat + Ash content)].

The protein content, measured at 2.35% for the KC and 3.07% for KPP, suggests that the plantain peel and/or the kefir grains may contain nitrogen compounds. This could also be a result of the enhanced proteolytic activity of the kefir microorganisms during fermentation. This finding is consistent with studies that have incorporated plant residues into fermented beverages, demonstrating similar increases in protein content. The increase in protein is nutritionally desirable, as it contributes to the greater biological value of the product (14).

However, the carbohydrate content was reduced from 12.29% (KC) to 9.47% (KPP). This influenced the total energy value. The carbohydrate reduction may be related to the use of simple sugars by microorganisms and the dilution of the glycidic fraction caused by the increased presence of insoluble fibers in the peel. Increasing the concentration of fruit peel and the incubation time increases the activity of LAB, accelerating the breakdown of carbohydrates and resulting in a lower final carbohydrate content in the fermented beverage. Overall, incorporating plantain peel has proven to be a promising strategy for optimizing milk kefir, thereby promoting improvements in the nutritional value of this product (16, 61).

### 3.4 Microbial viability during storage (AAB, LAB and yeast)

**Figure 6:**
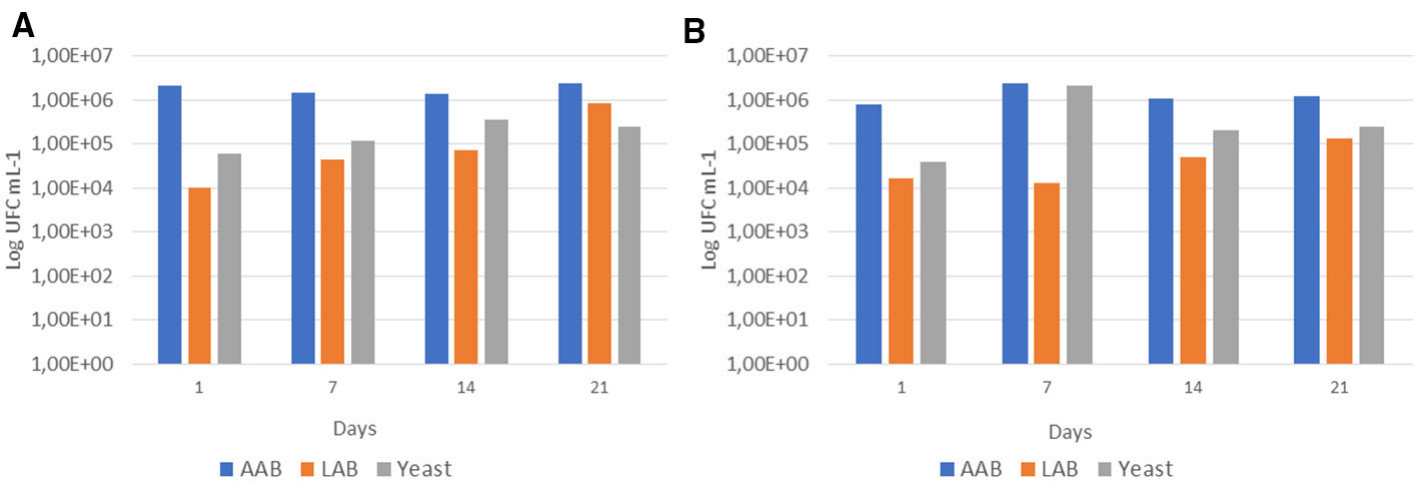
Microbial viability during 21 days of refrigerated storage, measured as log CFU ml^−1^ **(A)** Kefir-based Control (KC). **(B)** Optimized Kefir-based Beverage (KPP).

Acetic acid bacteria (AAB) and Yeast dominate the beverage microbiota compared to lactic acid bacteria (LAB). On the first day of storage, KC exhibited acetic acid bacteria at ~10^6^ CFU ml^−1^ and KPP at ~10^5^ CFU ml^−1^, with counts remaining stable at 10^6^ CFU ml^−1^ during 21 days of cold storage. In contrast, yeast counts in the control beverage remained low and relatively stable throughout storage, with a higher count at T14 (4.5 × 105 CFU ml^−1^). Conversely, KPP displayed a peak at T7 (~2.0 × 106 CFU ml^−1^), followed by a decline at later time points (~3–4 × 105 CFU ml^−1^). LAB was not detected during the first 7 days, and at 21 days of storage, appeared at higher levels in KC than in KPP (Figures 6A, B).

Several studies report that LAB counts in milk kefir are initially high after fermentation but may decrease or show limited apparent growth during the initial days of refrigerated storage. This early stagnation or reduction is likely due to cold-induced metabolic slowdown, post-acidification stress, and possible cell injury from low temperatures, which can temporarily reduce cultivability on MRS agar, the standard medium for LAB enumeration (62–64).

However, the main reasons include detection limits of methods, medium suitability, pH changes, and microbial competition, especially overgrowth by yeasts and acetic acid bacteria. LAB may be present at low levels early in fermentation, below the detection threshold of standard culture-based methods. More sensitive techniques like quantitative PCR can detect LAB earlier, but traditional plating may miss them until their populations increase significantly (65).

By days 14–21, LAB counts often stabilize or increase, reaching ~10^4^ CFU ml^−1^, as observed in this study. The specific LAB species present (e.g., *Lactobacillus kefiranofaciens, Lactococcus lactis*) and the initial fermentation conditions can influence these dynamics (64). The dietary fiber content contributed by fruit waste can be consumed as a prebiotic supplement to promote the survival rate and metabolic action of probiotics, such as *Lactobacillus* spp. and *Bifidobacterium* spp (66). However, in this study, the addition of 20% green plantain peels did not demonstrate a detectable prebiotic effect on the kefir microorganisms, as a prebiotic effect is characterized by an increase in viable counts (67).

Kefir grains contain live bacteria and yeasts that continue to metabolize sugars and produce acids, gases, and alcohol even after fermentation is complete. This ongoing activity can lead to increased acidity, texture changes, and off-flavors during storage, particularly over 21 days (68). Fermentation dynamics reflect complex, time-dependent interactions among microbial communities, metabolite production, and environmental factors. AAB produces acetic acid, while LAB plays a central role in acidification through lactic acid production. Overall process outcomes are shaped by microbial succession, substrate changes, and external conditions. Understanding these dynamics is key to optimizing fermentation to improve quality, safety, and process efficiency (69).

### 3.5 Microbiological safety

The samples were considered safe for consumption, with the absence of *Salmonella* spp and *E. coli* < 10 CFU ml^−1^ (36). Studies show that kefir beverages with pH maintained below 4.5 consistently support high counts of beneficial bacteria and yeasts, while creating an environment hostile to many spoilage and pathogenic bacteria, reducing the risk of contamination and spoilage during storage (70, 71).

### 3.6 Sensory evaluation

There were 114 participants in the sensory acceptance test: 75.4% female and 24.6% male. 40% of participants have never tried a kefir-based beverage or have tried it less than once a month. Meanwhile, 28% consume milk kefir once a week, and 24.6% consume it two to four times a week. Only 7% of participants consume kefir once a day. Daily kefir consumption is uncommon in Brazil. Surveys indicate that kefir is less frequently consumed than other fermented milk drinks like yogurt. In a study of 271 consumers, the majority reported drinking kefir and buttermilk less than once a week, while yogurt was consumed 3–5 times per week by 40% of respondents. Large-scale Russian dietary surveys also show that average kefir intake is relatively low, increasing from 10.9 to 25.6 grams per day over two decades, with higher consumption among older adults (72, 73).

**Figure 7:**
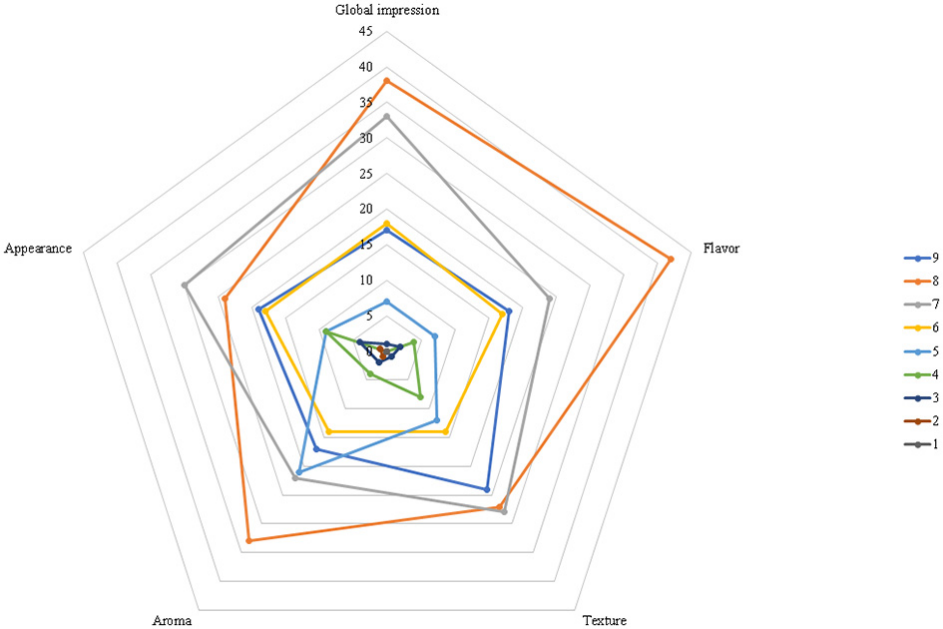
Sensory profile (spider web diagram) of the optimized kefir-based beverage (KPP) evaluated by 114 untrained tasters using a nine-point hedonic scale.

The overall impression attribute had a mean score of 7.32 ± 1.18 points on the hedonic scale (Figure 7), corresponding to an 81.29% acceptance index (AI). All attributes achieved acceptability indices above 70%, a level considered satisfactory for food products (37). These results indicate that the addition of green plantain peel to milk kefir maintained good sensory acceptance, especially in the overall attribute, flavor, and texture, suggesting potential for the development of a differentiated product acceptable. However, the aroma and appearance attributes, although acceptable, had slightly lower mean scores, which may reflect two main factors: first, plantain peel can introduce shades or color variations that not all evaluators find pleasant; second, volatile compounds associated with the peel or fermentation process may affect aroma, especially if they are not completely dominant or well-integrated into the sensory profile of kefir.

Comparing with the study by Contim et al. (74), which evaluated kefir with graviola pulp, high acceptability indices were also observed, with flavor and texture scoring well, while appearance and aroma had greater variations among evaluators. In the study of the effect of yacon potato on kefir acceptability (75), appearance and aroma were well-accepted, but flavor was close to the indifference threshold in some formulations, indicating that vegetable/tuberous additions require adjustments to balance flavor and aroma.

**Figure 8:**
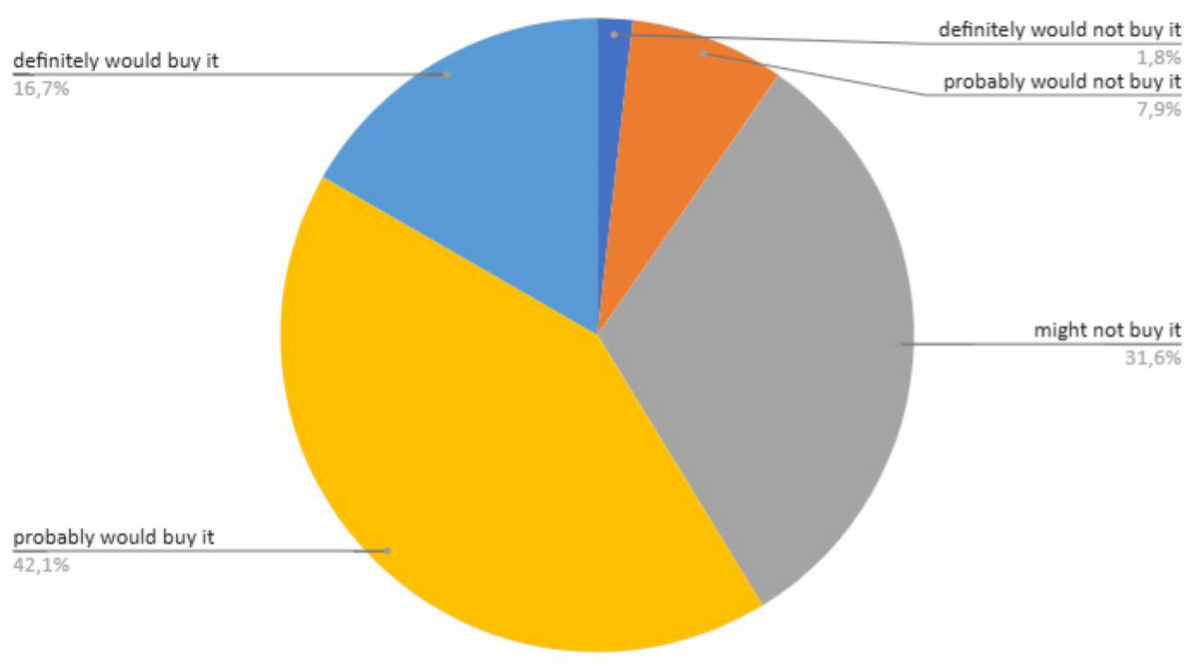
Evaluation of consumer purchase intention for the optimized kefir-based beverage (KPP; *n* = 114) using a structured five-point scale. based beverage (KPP; *n* = 114).

Regarding purchase intention, 58.8% of consumers indicated they would buy the product (Figure 8). The optimal formulation of the kefir-based beverage containing green plantain peel (GPP) indicates favorable characteristics for market acceptance, showing good purchase intention.

## 4 Conclusion

The optimized fermentation conditions, based on response surface methodology, were 20% green plantain peel and 10% kefir grains. The incorporation of 20% green plantain peel into milk kefir promoted relevant changes in the proximal composition and the total phenolic compounds of the beverage. The optimized formulation showed higher moisture and protein contents, suggesting improved water retention due to soluble fibers and enhanced of microbial proteolysis. The microbial viability (LAB and yeasts) was maintained during refrigerated storage. Lipid and ash contents remained stable, while carbohydrate concentration decreased, leading to a lower caloric value. The significantly higher firmness of the optimized formulation suggests improved textural stability and maintained a favorable mouthfeel, contributing to the high overall acceptability index of 81.29% in sensory analysis. These modifications indicate a nutritionally and sensorially advantageous profile, characterized by higher protein density and reduced energy contribution, reinforcing the potential of plantain peel as a sustainable ingredient to improve the nutritional and functional quality of fermented dairy beverages.

## Data Availability

The raw data supporting the conclusions of this article will be made available by the authors, without undue reservation.

## References

[1] Paredes JL Escudero-Gilete ML Vicario IM. A new functional kefir fermented beverage obtained from fruit and vegetable juice: development and characterization. LWT. (2022) 154:112728. doi: 10.1016/j.lwt.2021.112728

[2] Khan I Nadeem M Imran M Ullah R Ajmal M Jaspal M. Antioxidant properties of milk and dairy products: a comprehensive review of the current knowledge. Lipids Health Dis. (2019) 18:14. doi: 10.1186/s12944-019-0969-8 30717735 PMC6362592

[3] Górska-Warsewicz H Rejman K Laskowski W Czeczotko M. Milk and dairy products and their nutritional contribution to the average polish diet. Nutrients. (2019) 11:1771. doi: 10.3390/nu11081771 31374893 PMC6723869

[4] Stachelska M Karpiński P Kruszewski B. Health-promoting and functional properties of fermented milk beverages with probiotic bacteria in the prevention of civilization diseases. Nutrients. (2024) 17:9. doi: 10.3390/nu17010009 39796443 PMC11722897

[5] Ballini A Charitos I Cantore S Topi S Bottalico L Santacroce L. About functional foods: the probiotics and prebiotics state of art. Antibiotics. (2023) 12:635. doi: 10.3390/antibiotics12040635 37106999 PMC10135203

[6] Peres AP Domingues YO Beserra BTS Guimarães NS Bento JAC Porto YD . The consumption of milk or dairy products and sleep quality: a systematic review and meta-analysis. Cureus. (2025) 17:e92556. doi: 10.7759/cureus.92556 41111656 PMC12533936

[7] Silva R Mancuso M. Marcadores identitários mato-grossenses: a comida nos rasqueados. Revista Inter-Legere. (2019) 2:c17376-c. Portuguese. doi: 10.21680/1982-1662.2019v2n25ID17376

[8] Amini Khoozani A Birch J Bekhit AE-DA. Production, application and health effects of banana pulp and peel flour in the food industry. J Food Sci Technol. (2019) 56:548–59. doi: 10.1007/s13197-018-03562-z 30906012 PMC6400781

[9] Martharini D Indratiningsih I. Microbiological and chemical quality of goat milk kefir with the addition of *Lactobacillus acidophilus* FNCC 0051 and plantain peel flour (*Musa paradisiaca*). Agritech Jurnal Teknologi Pertanian. (2017) 37:22–9. doi: 10.22146/agritech.17002

[10] Storck CR Nunes GL Oliveira BBd Basso C. Folhas, talos, cascas e sementes de vegetais: composição nutricional, aproveitamento na alimentação e análise sensorial de preparações. Ciência Rural. (2013) 43:537–43. Portuguese. doi: 10.1590/S0103-84782013000300027

[11] Gad A Orabi M Abou-Taleb K Abdelghani D Amin S. *In vitro* digestive system simulation and anticancer activity of soymilk fermented by probiotics and synbiotics immobilised on agro-industrial residues. Sci Rep. (2024) 14:18518. doi: 10.1038/s41598-024-68086-3 39122808 PMC11316043

[12] Neta MCA Queiroga APR Almeida R Soares AC Gonçalves JM Fernandes SS . Fermented dessert with whey, ingredients from the peel of Jabuticaba (Myrciaria cauliflora) and an indigenous culture of *Lactobacillus plantarum*: composition, microbial viability, antioxidant capacity and sensory features. Nutrients. (2018) 10:1214. doi: 10.3390/nu10091214 30200532 PMC6163542

[13] Sousa MC Santos WMD Da Silva JMO Ramos FP Freitas AS Neta MCA . Non-fermented dairy desserts with potentially probiotic autochthonous lactobacilli and products from peel of Jabuticaba (Myrciaria cauliflora). Probiot Antimicrob Proteins. (2021) 13:1–11. doi: 10.1007/s12602-020-09731-x 33404867

[14] Vicenssuto GM De Castro RJS. Development of a novel probiotic milk product with enhanced antioxidant properties using mango peel as a fermentation substrate. Biocatal Agric Biotechnol. (2020) 24. doi: 10.1016/j.bcab.2020.101564

[15] Silva ARS Peres AP Martins RAdS Magalhães KT Puerari C Morzelle MC . The addition of plantain peel (*Musa paradisiaca*) to fermented milk as a strategy for enriching the product and reusing agro-industrial waste. Beverages. (2025) 11:153. doi: 10.3390/beverages11050153

[16] Oliveira FLd Arruda TYP Morzelle MC Pereira APA Casarotti SN. Fruit by-products as potential prebiotics and promising functional ingredients to produce fermented milk. Food Res Int. (2022) 161:111841. doi: 10.1016/j.foodres.2022.111841 36192971

[17] FAO. Think Eat Save - Tracking Progress to Halve Global Food Waste. Rome: Food and Agriculture Organization of the United Nations (2024). Available online at: https://www.fao.org/faostat/en/ (Accessed 2024).

[18] Gupta RK Ali EA Gawad FAE Daood VM Sabry H Karunanithi S . Valorization of fruits and vegetables waste byproducts for development of sustainable food packaging applications. Waste Manag Bulletin. (2024) 24. doi: 10.1016/j.wmb.2024.08.005

[19] Putra NR Aziz AH Faizal AN Che Yunus MA. Methods and potential in valorization of banana peels waste by various extraction processes: in review. Sustainability. (2022) 14:10571. doi: 10.3390/su141710571

[20] Von Loesecke HW. Bananas. (1949).

[21] Feitosa VBD Oliveira ENAd Souza RLAd Feitosa BF Feitosa RM. Estabilidade físico-química de iogurtes adoçados com mel de abelha *Apis mellifera* L. Ciência Animal Brasileira. (2020) 21:e-50923. Portuguese. doi: 10.1590/1809-6891v21e-50923

[22] Ministério da Agricultura PeAM. Instrução Normativa n° 16, de 23 de agosto de 2005. Brasilia: MAPA (2005). Portuguese

[23] ISO. Fermented Milks — Determination of Titratable Acidity — Potentiometric Method. Geneva: ISO (2012).

[24] Woisky RG Salatino A. Analysis of propolis: some parameters and procedures for chemical quality control. J Apic Res. (1998) 37:99–105. doi: 10.1080/00218839.1998.11100961

[25] Brand-Williams W Cuvelier M-E Berset C. Use of a free radical method to evaluate antioxidant activity. LWT Food Sci Technol. (1995) 28:25–30. doi: 10.1016/S0023-6438(95)80008-5

[26] Nenadis N Wang L-F Tsimidou M Zhang H-Y. Estimation of scavenging activity of phenolic compounds using the ABTS•+ assay. J Agric Food Chem. (2004) 52:4669–74. doi: 10.1021/jf0400056 15264898

[27] Pulido R Bravo L Saura-Calixto F. Antioxidant activity of dietary polyphenols as determined by a modified ferric reducing/antioxidant power assay. J Agric Food Chem. (2000) 48:3396–402. doi: 10.1021/jf9913458 10956123

[28] Mohamed-Ahmed IA Alqah HAS Saleh A Al-Juhaimi FY Babiker EE Ghafoor K . Physicochemical quality attributes and antioxidant properties of set-type yogurt fortified with argel (Solenostemma argel Hayne) leaf extract. LWT. (2021) 137:110389. doi: 10.1016/j.lwt.2020.110389

[29] Mohamed-Ahmed IA Özcan MM Al Juhaimi F Babiker EFE Ghafoor K Banjanin T . Chemical composition, bioactive compounds, mineral contents, and fatty acid composition of pomace powder of different grape varieties. J Food Process Preserv. (2020) 44:e14539. doi: 10.1111/jfpp.14539

[30] Liu Y-H MacFie H. Methods for averaging time–intensity curves. Chem Senses. (1990) 15:471–84. doi: 10.1093/chemse/15.4.471

[31] AOAC I. Official Methods of Analysis of AOAC International. 16th ed. Arlington, VA: AOAC International (1995).

[32] Wisker E Feldheim W. Metabolizable energy of diets low or high in dietary fiber from fruits and vegetables when consumed by humans. J Nutr. (1990) 120:1331–7. doi: 10.1093/jn/120.11.1331 2172491

[33] Bongaerts D Bouchez A De Roos J Cnockaert M Wieme AD Vandamme P . Refermentation and maturation of lambic beer in bottles: a necessary step for gueuze production. Appl Environ Microbiol. (2024) 90:e01869–23. doi: 10.1128/aem.01869-23 38446583 PMC11022581

[34] Tavares P Nascimento E Cruz R Lemos L Druzian P Izabel J . Chemical, microbiological and sensory viability of low-calorie, dairy-free kefir beverages from tropical mixed fruit juices. CyTA J Food. (2021) 19:457–64. doi: 10.1080/19476337.2021.1906753

[35] Ministério da Agricultura PeAM. Instrução Normativa n° 161, de 1° de julho de 2022. Brasilia: MAPA (2022). Portuguese.

[36] ISO. Microbiology of Food and Animal Feeding Stuffs — Horizontal Method for the Detection of Salmonella spp. Geneva: ISO (2002).

[37] Dutcosky SD. Análise Sensorial de Alimentos. Ampliada ere, editor. Exatas (2019). Curitiba: Portuguese.

[38] Coelho M Malcata F Silva C. Lactic acid bacteria in raw-milk cheeses: from starter cultures to probiotic functions. Foods. (2022) 11:2276. doi: 10.3390/foods11152276 35954043 PMC9368153

[39] Adi DD Oduro IN Tortoe C. Physicochemical changes in plantain during normal storage ripening. Sci African. (2019) 6:e00164. doi: 10.1016/j.sciaf.2019.e00164

[40] Inada KOP Silva TBR Lobo LA Domingues RMCP Perrone D Monteiro M. Bioaccessibility of phenolic compounds of jaboticaba (*Plinia jaboticaba*) peel and seed after simulated gastrointestinal digestion and gut microbiota fermentation. J Function Foods. (2020) 67:103851. doi: 10.1016/j.jff.2020.103851

[41] Ortega-Hernández E Martinez-Alvarado L Acosta-Estrada BA Antunes-Ricardo M. Solid-state fermented pineapple peel: a novel food ingredient with antioxidant and anti-inflammatory properties. Foods. (2023) 12:4162. doi: 10.3390/foods12224162 38002219 PMC10670571

[42] Tsamo CVP Herent M-F Tomekpe K Emaga TH Quetin-Leclercq J Rogez H . Phenolic profiling in the pulp and peel of nine plantain cultivars (Musa sp). Food Chem. (2015) 167:197–204. doi: 10.1016/j.foodchem.2014.06.095 25148979

[43] Sabokbar N Khodaiyan F. Total phenolic content and antioxidant activities of pomegranate juice and whey based novel beverage fermented by kefir grains. J Food Sci Technol. (2015) 53:739–47. doi: 10.1007/s13197-015-2029-3 26787994 PMC4711455

[44] Sabokbar N Khodaiyan F Moosavi-Nasab M. Optimization of processing conditions to improve antioxidant activities of apple juice and whey based novel beverage fermented by kefir grains. J Food Sci Technol. (2014) 52:3422–32. doi: 10.1007/s13197-014-1397-4 26028723 PMC4444860

[45] Viogenta P Khairunnisa A Kartinah N Rahmadati E. Antioxidant assay of kefir peanut (*Arachis hypogaea* L.) with variations in concentration and fermentation time. Majalah Obat Tradisional. (2024) 29. doi: 10.22146/mot.87814

[46] El-Katony T El-Dein N El-Fallal A Ibrahim N Mousa M. Substrate–fungus interaction on the enzymatic and non-enzymatic antioxidant activities of solid state fermentation system. Bioresour Bioprocess. (2020) 7:1–11. doi: 10.1186/s40643-020-00316-8

[47] Prado MR Blandón LM Vandenberghe LP Rodrigues C Castro GR Thomaz-Soccol V . Milk kefir: composition, microbial cultures, biological activities, and related products. Front Microbiol. (2015) 6:1177. doi: 10.3389/fmicb.2015.01177 26579086 PMC4626640

[48] Rosa DD Dias MM Grześkowiak ŁM Reis SA Conceição LL Peluzio MdCG. Milk kefir: nutritional, microbiological and health benefits. Nutr Res Rev. (2017) 30:82–96. doi: 10.1017/S0954422416000275 28222814

[49] Yilmaz-Ersan L Ozcan T Akpinar-Bayizit A Sahin S. Comparison of antioxidant capacity of cow and ewe milk kefirs. J Dairy Sci. (2018) 101:3788–98. doi: 10.3168/jds.2017-13871 29477522

[50] Lagouri V Dimitreli G Kouvatsi A. Effects of Greek pomegranate extracts in the antioxidant properties and storage stability of kefir. Curr Bioact Compd. (2019) 15. doi: 10.2174/1573407214666180808113450

[51] Liu Y Chen H Chen W Zhong Q Zhang G Chen W. Beneficial effects of tomato juice fermented by *Lactobacillus plantarum* and *Lactobacillus casei*: antioxidation, antimicrobial effect, and volatile profiles. Molecules. (2018) 23:2366. doi: 10.3390/molecules23092366 30223615 PMC6225183

[52] Magalhães KT Pereira GVdM Campos CR Dragone G Schwan RF. Brazilian kefir: structure, microbial communities and chemical composition. Braz J Microbiol. (2011) 42:693–702. doi: 10.1590/S1517-83822011000200034 24031681 PMC3769826

[53] Xiao R Liu M Tian Q Hui M Shi X Hou X. Physical and chemical properties, structural characterization and nutritional analysis of kefir yoghurt. Front Microbiol. (2023) 13:1107092. doi: 10.3389/fmicb.2022.1107092 36713216 PMC9874054

[54] Wulansari P Widodo Sunarti Nurliyani. Incorporation of oat milk with probiotic *Lacticaseibacillus casei* AP improves the quality of kefir produced from goat milk. Food Sci Technol. (2022) 42. doi: 10.1590/fst.10322

[55] Biçer Y Turkal G Sönmez G Telli AE Bayir T Çulha MH . Production of yoghurt from kefir beverage: analysis of fermentation kinetics, volatile organic compounds, texture, and microbial characteristics. Int Dairy J. (2024) 158:106039. doi: 10.1016/j.idairyj.2024.106039

[56] Safdari Y Vazifedoost M Didar Z Hajirostamloo B. The effect of banana fiber and banana peel fiber on the chemical and rheological properties of symbiotic yogurt made from camel milk. Int J Food Sci. (2021) 2021:5230882. doi: 10.1155/2021/5230882 34957296 PMC8695018

[57] Agama-Acevedo E Sañudo-Barajas J Rocha R González-Aguilar G Bello-Pérez L. Potential of plantain peels flour (*Musa paradisiaca* L) as a source of dietary fiber and antioxidant compound. CyTA J Food. (2016) 14:117–23. doi: 10.1080/19476337.2015.1055306

[58] Gaya LG Ferraz JBS. Aspectos genético-quantitativos da qualidade da carne em frangos. Ciência Rural. (2006) 36:349–56. Portuguese. doi: 10.1590/S0103-84782006000100058

[59] García-Valle D Bello-Pérez L Flores-Silva P Agama-Acevedo E Tovar J. Extruded unripe plantain flour as an indigestible carbohydrate-rich ingredient. Front Nutr. (2019) 6:2. doi: 10.3389/fnut.2019.00002 30805343 PMC6370669

[60] Çoşkun F Erol H. The quality of kefir with honey and with banana enriched with almond milk. Turk J Agri Food Sci Technol. (2023) 11. doi: 10.24925/turjaf.v11i8.1337-1344.6007

[61] De Souza HF Monteiro GF Bogáz LT Freire ENS Pereira K De Carvalho MV . Bibliometric analysis of water kefir and milk kefir in probiotic foods from 2013 to 2022: a critical review of recent applications and prospects. Food Res Int. (2023) 175:113716. doi: 10.1016/j.foodres.2023.113716 38128984

[62] Leite A Leite D Águila E Alvares T Peixoto R Miguel M . Microbiological and chemical characteristics of Brazilian kefir during fermentation and storage processes. J Dairy Sci. (2013) 7:4149–59. doi: 10.3168/jds.2012-6263 23628252

[63] Montanuci F Pimentel T Garcia S Prudêncio S. Effect of starter culture and inulin addition on microbial viability, texture, and chemical characteristics of whole or skim milk Kefir. Food Sci Technol Int. (2012) 32:850–61. doi: 10.1590/S0101-20612012005000119

[64] Güney HD Altundag ÖÖ Çolak M. Microbiological changes of kefir traditionally produced from different milks according to storage time. Front Life Sci Relat Technol. (2025) 6. doi: 10.51753/flsrt.1561917

[65] Gamba R Koyanagi T Peláez Á De Antoni G Enomoto T. Changes in microbiota during multiple fermentation of kefir in different sugar solutions revealed by high-throughput sequencing. Curr Microbiol. (2021) 78:2406–13. doi: 10.1007/s00284-021-02501-0 33961093

[66] Akter B Rabeta M. Symbiotic and antioxidant activity of fruit by-products and their effect on human health. Food Res. (2021) 5:24–35. doi: 10.26656/fr.2017.5(1)0.401

[67] Mahomud MS Islam MN Hossen D Wazed MA Yasmin S Sarker MSH. Innovative probiotic yogurt: leveraging green banana peel for enhanced quality, functionality, and sensory attributes. Heliyon. (2024) 10:e38781. doi: 10.1016/j.heliyon.2024.e38781 39421385 PMC11483293

[68] Irigoyen A Arana I Castiella M Torre P Ibanez F. Microbiological, physicochemical, and sensory characteristics of kefir during storage. Food Chem. (2005) 90:613–20. doi: 10.1016/j.foodchem.2004.04.021

[69] Moimenta AR Troitiño-Jordedo D Henriques D Contreras-Ruíz A Minebois R Morard M . An integrated multiphase dynamic genome-scale model explains batch fermentations led by species of the *Saccharomyces genus*. Msystems. (2025) 10:e01615–24. doi: 10.1128/msystems.01615-24 39840996 PMC11838008

[70] Limbad M Gutierrez-Maddox N Hamid N Kantono K Liu T Young T. Microbial and chemical changes during fermentation of coconut water kefir beverage. Appl Sci. (2023) 13:7257. doi: 10.3390/app13127257

[71] Silva JCdM Santana RV Almeida ABd Takeuchi KP Egea MB. Changes in the chemical, technological, and microbiological properties of kefir-fermented soymilk after supplementation with inulin and acrocomia aculeata pulp. Appl Sci. (2021) 11:5575. doi: 10.3390/app11125575

[72] Martinchik A Baturin A Keshabyants E Mihaylov N Picard-Deland É Marette A. Fermented dairy products consumption and impact on nutrients intake and nutritional status by anthropometric data in Russian adults: RLMS-HSE. FASEB J. (2015) 29:734.6. doi: 10.1096/fasebj.29.1_supplement.734.6

[73] Sawa A Feldheim J Kreżel-Czopek S. Frequency of consumption of fermented milk drinks and factors influencing consumer Choice. Acta Sci Pol Zootech. (2018) 17. doi: 10.21005/asp.2018.17.2.04

[74] Contim LSR Oliveira IMA Cardoso Neto J. Microbiological, Physicochemical Evaluation and Sensory Acceptance of Kefir With Graviola Pulp. (Lepidus Tecnologia). (2018). doi: 10.14295/2238-6416.v73i1.604

[75] Gonçalves IF Martins EMF Silva VRO de Oliveira Martins AD. Efeito de yacon na aceitação sensorial de kefir e viabilidade de bactérias láticas na bebida. Vértices. (2018) 20:194–201. Portuguese. doi: 10.19180/1809-2667.v20n22018p194-201

